# Robust ferromagnetism in hydrogenated graphene mediated by spin-polarized pseudospin

**DOI:** 10.1038/s41598-018-31934-0

**Published:** 2018-09-17

**Authors:** Hyunyoung Kim, Junhyeok Bang, Joongoo Kang

**Affiliations:** 1 Department of Emerging Materials Science, DGIST, Daegu, 42988 Korea; 2 Spin Engineering Physics Team, Korea Basic Science Institute (KBSI), Daejeon, Korea; 3 Center for Bio-Convergence Spin System, DGIST, Daegu, 42988 Korea

## Abstract

The origin of the ferromagnetism in metal-free graphitic materials has been a decade-old puzzle. The possibility of long-range magnetic order in graphene has been recently questioned by the experimental findings that point defects in graphene, such as fluorine adatoms and vacancies, lead to defect-induced paramagnetism but no magnetic ordering down to 2 K. It remains controversial whether collective magnetic order in graphene can emerge from point defects at finite temperatures. This work provides a new framework for understanding the ferromagnetism in hydrogenated graphene, highlighting the key contribution of the spin-polarized pseudospin as a “mediator” of long-range magnetic interactions in graphene. Using first-principles calculations of hydrogenated graphene, we found that the unique ‘zero-energy’ position of H-induced quasilocalized states enables notable spin polarization of the graphene’s sublattice pseudospin. The pseudospin-mediated magnetic interactions between the H-induced magnetic moments stabilize the two-dimensional ferromagnetic ordering with Curie temperatures of T_c_ = n_H_ × 34,000 K for the atom percentage n_H_ of H adatoms. These findings show that atomic-scale control of hydrogen adsorption on graphene can give rise to a robust magnetic order.

## Introduction

The two-dimensional (2D) magnetism in graphene has attracted considerable attention because of its exceptional promise in graphene-based spintronics^1–12^. Several experiments showed that the ferromagnetic order in graphitic materials originates from the carbon *π*-electron systems rather than from magnetic impurities^13–15^. Further studies have shown that point defects in graphitic materials contribute to carbon-based ferromagnetism^16–21^. Recently, proximity-induced ferromagnetism^22–25^ was also demonstrated for a single-layer graphene placed on an insulating magnetic substrate. Despite decades of research on carbon-based magnetism, it remains unclear under what conditions long-range magnetic order can emerge from point defects in graphitic materials. Contrary to the previous notion that graphene ferromagnetism arises from defect-induced magnetic moments^16–21^, Nair *et al*.^5^ recently demonstrated that point defects in graphene, such as fluorine adatoms and vacancies, lead to notable paramagnetism but no magnetic ordering down to liquid helium temperatures. The maximum response of the induced paramagnetism was limited to one moment per approximately 1,000 carbon atoms. The lack of collective magnetic order in graphene was attributed to the absence of long-range magnetic interactions between the well-separated magnetic moments^5^, with the implication that previously reported room-temperature ferromagnetism in graphitic materials^3,13–15,26,27^ might originate from undetected magnetic impurities or particles.

Recent scanning tunneling microscope experiments^11^ have provided direct evidence that individual hydrogen atoms adsorbed on graphene induce magnetic moments, creating opportunities for atomic-scale control of graphene ferromagnetism^11,12^. As in the graphene systems with fluorine adatoms or carbon vacancies^5^, however, the magnetic response in hydrogenated graphene is limited because the phase separation into pure graphene and fully hydrogenated graphene (called graphane) parts is thermodynamically more stable^28^. The ferromagnetism at reasonably high temperatures in such a magnetically dilute system thus requires long-range magnetic interactions between the H-induced magnetic moments, as well as controlled hydrogenation of graphene under non-equilibrium conditions.

**Figure 1:**
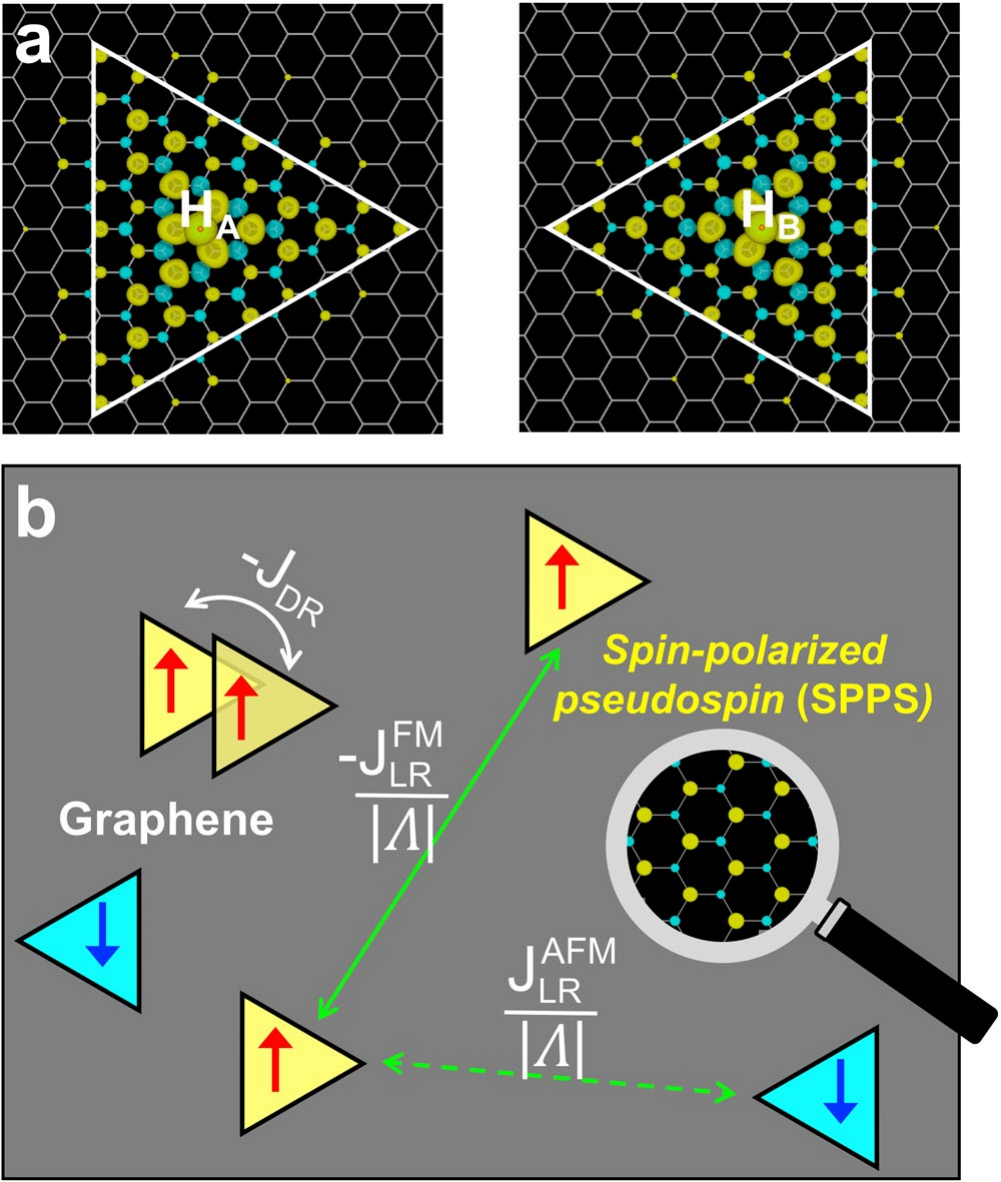
Direct versus “mediated” magnetic interactions in hydrogenated graphene. (**a**) The localized magnetic moments generated by an H adatom at the center: from left to right, an H adatom at an A sublattice site and an H adatom at a B sublattice site of the graphene. The electron density is shown for the spin-up (yellow) and spin-down (blue) electrons at 0.0027 |*e*|/Å^3^. (**b**) Schematic illustration of two different types of magnetic interaction between the localized spins in hydrogenated graphene: the direct (DR) exchange interaction, *J*_DR_, which arises from the overlap between the localized wavefunctions (represented by triangles), and the long-range (LR) magnetic interaction, $\frac{{J}_{LR}^{FM}}{|{\rm{\Lambda }}|}$ or $\frac{{J}_{LR}^{AFM}}{|{\rm{\Lambda }}|}$, mediated by the spin-polarized pseudospin of graphene (zoomed-in view).

Using spin-polarized density functional theory (DFT) calculations, we show that hydrogenated graphene not only hosts H-induced localized spins but also responds to them by forming spin-polarized pseudospin as a “mediator” of long-range magnetic interactions in graphene. In hydrogenated graphene, it is well-known that the C–H *σ*-bond formation effectively induces a “vacancy” in the *π*-electron system^2,29–31^, creating a quasilocalized state occupied by an electron^11,32,33^ and an associated magnetic moment (Fig. 1a). The H-induced ‘vacancy’ state lies almost at the Dirac-point energy^11,30,31^, unlike the cases of fluorine adatoms and carbon vacancies with the corresponding defect state at lower energy^2,30^. The unique energy position of the H-induced ‘vacancy’ state in the half-filled *π*-electron system enables strong pseudospin-mediated magnetic interactions. Hereafter, we refer to the H-induced “vacancy” state as an “A-vacancy” or “B-vacancy”, depending on which sublattice of the graphene contains the adsorption site.

## Results and Discussion

### Direct versus “mediated” magnetic interactions in hydrogenated graphene

**Figure 2:**
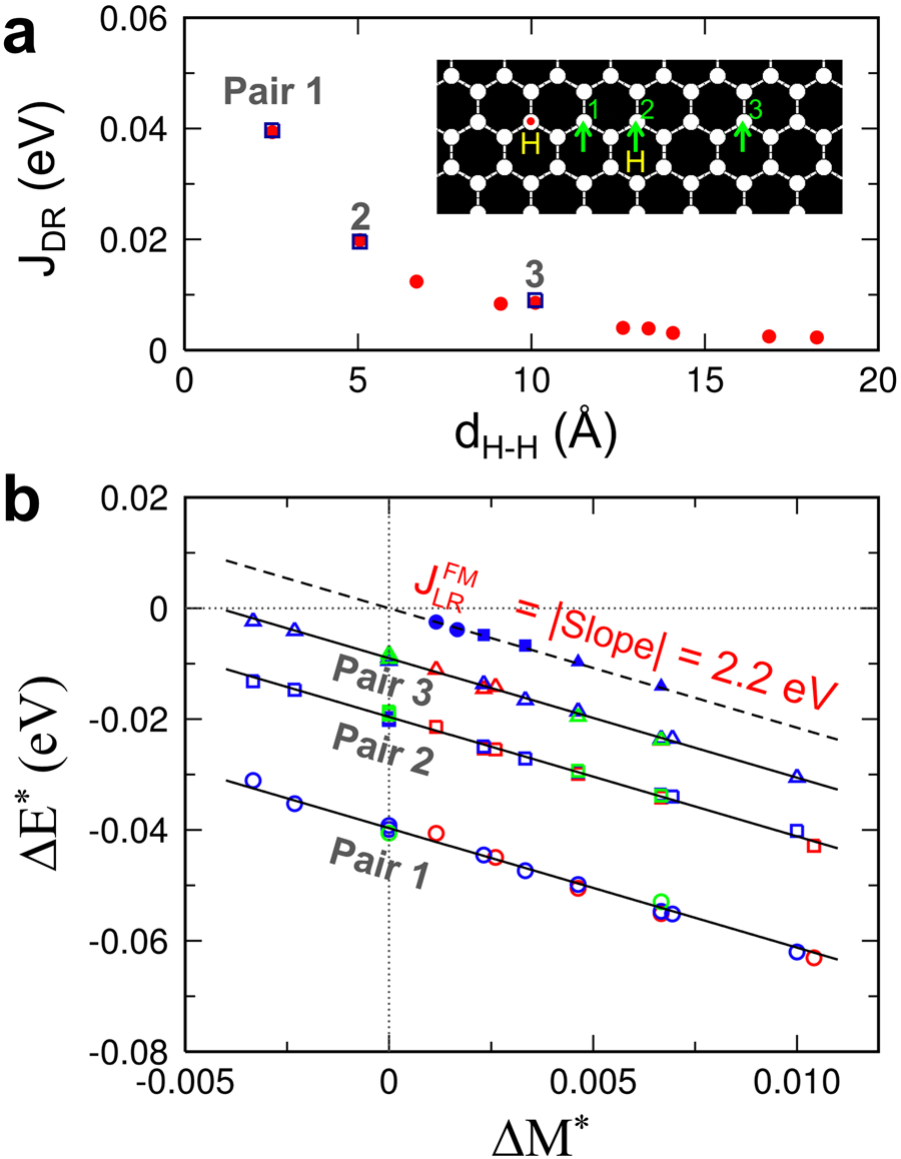
Distinguishing the long-range “mediated” interaction from the direct exchange interaction. (**a**) The direct exchange interaction *J*_DR_ as a function of the H–H distance (*d*_H-H_). (**b**) Extracting the long-range “mediated” interaction ${J}_{LR}^{{\rm{FM}}}$ from the linear relation between ∆M^*^ and ∆E^*^ in Eq. 2. The selected H pairs, labeled 1, 2, and 3, are shown in the inset of (**a**). For a given pair type, the DFT data lie almost on a straight line with a slope of $-{J}_{LR}^{{\rm{FM}}}$, and the absolute value of the y-intercept corresponds to ${J}_{DR}^{{\rm{pair}}}$. The fitted ${J}_{DR}^{{\rm{pair}}}$ values for the selected H pairs are shown as open squares in (**a**).

Figure 1b depicts two different types of magnetic interaction in hydrogenated graphene: (i) The direct (DR) exchange interaction J_DR_, which arises from the overlap between the wavefunctions of “vacancies”, leads to ferromagnetic (FM) interaction between “vacancies” in the same sublattices^11,18^. Because of the slow decay of the wavefunctions, the interaction range is relatively large (Fig. 2a). For two nearby “vacancies” in opposing sublattices (not shown in Fig. 1b), the electronic coupling between the “vacancy” states leads to short-range antiferromagnetic (AFM) interaction or even quenching of the defect-induced magnetic moments if the distance is too small^11,18,34^. (ii) In addition to the “conventional” magnetic interactions based on the wavefunction overlap, we show that “unconventional” long-range interactions exist that involve a delocalized “mediator” between the localized spins (zoomed-in view in Fig. 1b). The “mediator” (its physical nature will be discussed later) interacts with individual localized spins in a mean-field sense. The “mediated” magnetic interaction can be interpreted as a long-range pairwise interaction, whose strength is inversely proportional to the number of sites |*Λ*| in the graphene. The long-range (LR) interaction is FM for a pair of “vacancies” in the same sublattice $(\frac{{J}_{LR}^{FM}}{|{\rm{\Lambda }}|})$, while it is AFM for a pair in opposing sublattices $(\frac{{J}_{LR}^{AFM}}{|{\rm{\Lambda }}|})$.

To distinguish the long-range “mediated” magnetic interaction from the direct exchange interaction, we performed DFT calculations of hydrogenated graphene using judiciously chosen H-adatom positions (see Methods and Supplementary Table S1). The H adatoms in a graphene supercell exist either as an isolated H or as an H pair of a single type, which was chosen among the three types of pairs denoted by pair 1, 2, and 3 in the inset of Fig. 2a. The direct exchange interaction beyond 20 Å was neglected. First, we consider only the “A-vacancies”. The magnetic interaction energy is then given by

1 $$E=-\,\frac{{J}_{DR}^{pair}}{2}\sum _{(i,\,j)}{m}_{i}^{A}{m}_{j}^{A}-\frac{{J}_{LR}^{FM}}{2|{\rm{\Lambda }}|}\sum _{i,\,j}{m}_{i}^{A}{m}_{j}^{A},$$

where the symbol (*i*, *j*) in the first term denotes the H pairs of a given type, and ${J}_{DR}^{{\rm{pair}}}$ is the corresponding direct exchange interaction. A local magnetic moment ${m}_{i}^{A}$ at site *i* on the A sublattice can take either 1 (spin up) or –1 (down). The spin-polarized DFT calculations were performed in the S_z_ = S subspace; thus, $S=\frac{1}{2}| \sum _{i}{m}_{i}^{A}|$. We considered two different spin configurations, {*m*^(1)^} and {*m*^(2)^}, for which some of the FM-coupled (*i*, *j*) pairs in {*m*^(1)^} were changed to the AFM-coupled (*i*, *j*) pairs in {*m*^(2)^} (Supplementary Table S1). From the number of FM-to-AFM spin flips (*N*_flip_), the energy difference is given by ${E}^{(1)}-{E}^{(2)}=-\,2{N}_{{\rm{f}}{\rm{l}}{\rm{i}}{\rm{p}}}\,\,{J}_{DR}^{{\rm{p}}{\rm{a}}{\rm{i}}{\rm{r}}}-{J}_{LR}^{FM}\frac{{({\sum }_{i}{m}_{i}^{A(1)})}^{2}-{({\sum }_{i}{m}_{i}^{A(2)})}^{2}}{2|{\rm{\Lambda }}|}$. By defining ${\rm{\Delta }}{E}^{\ast }=\frac{{E}^{(1)}-{E}^{(2)}}{2{N}_{{\rm{flip}}}}$ and ${\rm{\Delta }}{M}^{\ast }=\frac{{({\sum }_{i}{m}_{i}^{A(1)})}^{2}-{({\sum }_{i}{m}_{i}^{A(2)})}^{2}}{4{N}_{{\rm{flip}}}|{\rm{\Lambda }}|}$, we obtain a simple linear relation between them:

2 $${\rm{\Delta }}{E}^{\ast }=-{J}_{DR}^{{\rm{pair}}}-{J}_{LR}^{FM}\,{\rm{\Delta }}{M}^{\ast }.$$

Indeed, the DFT data in Fig. 2b lie almost on straight lines with ${J}_{LR}^{FM}$ = 2.2 eV, which indicates that in addition to the direct exchange interaction (first term in Eq. 1), a long-range “mediated” interaction exists that is scaled with $\frac{1}{|{\rm{\Lambda }}|}$ (second term in Eq. 1).

### Long-range magnetic interactions mediated by spin-polarized pseudospin

**Figure 3:**
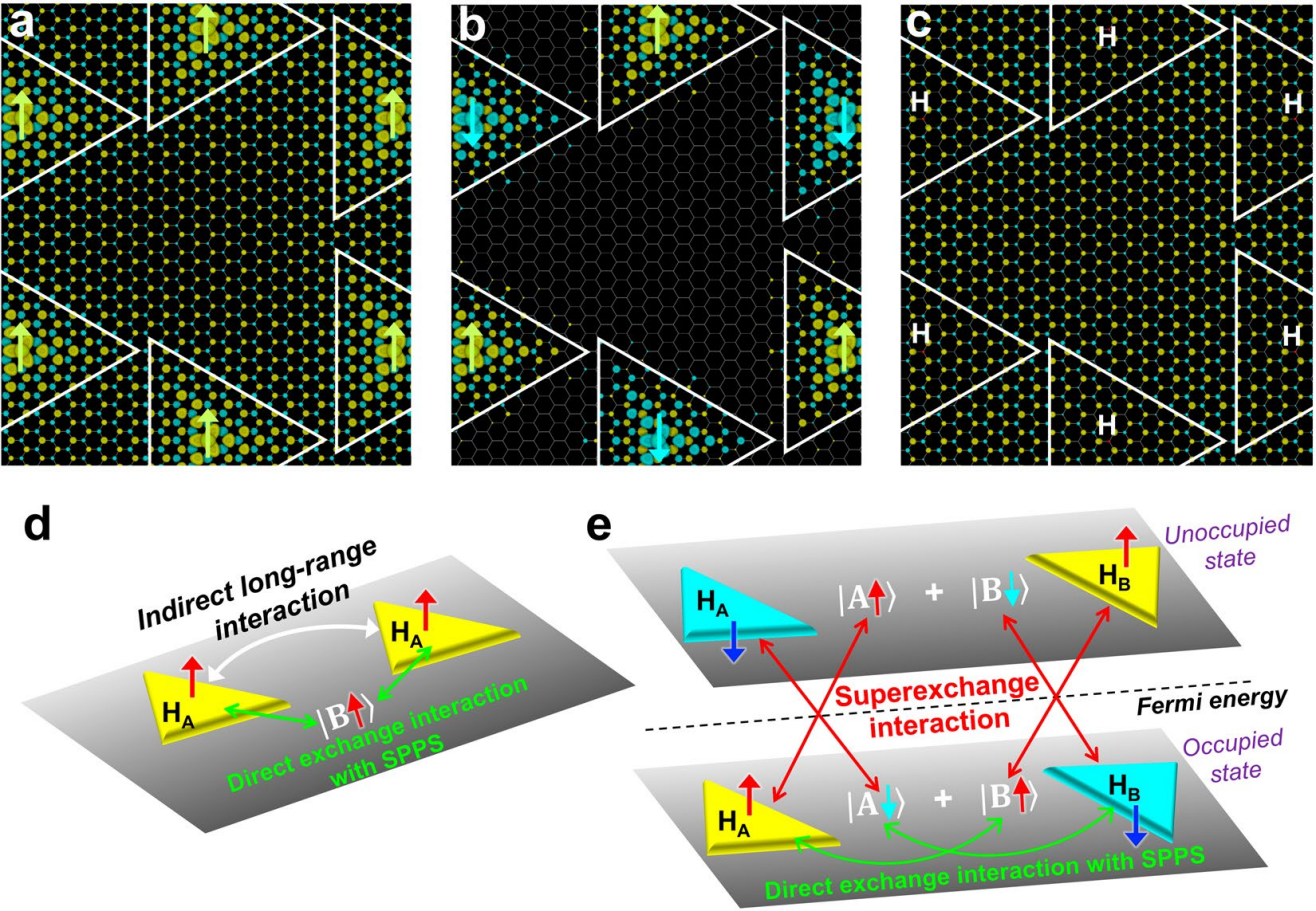
Long-range magnetic interactions mediated by spin-polarized pseudospin. (**a**,**b**,**c**) Spin densities of the 864-atom graphene supercells containing two H adatoms on the same sublattice. The spin density of the expanded supercell is shown for the spin-up (yellow) and spin-down (blue) electrons at 0.0016 |*e*|/Å^3^. In (**a**), two localized spins in a supercell are in the FM state, while in (**b**), they are in the AFM state. The spin-density plot in (**c**) reveals the delocalized spin-polarized pseudospin (SPPS), which is present only for (**a**). The SPPS was extracted from the results in (**a**) and (**b**) in two steps; we first flipped the spin values for the “spin-down” triangles in (**b**) and calculated the difference between the spin density in (**a**) and the modified density of (**b**) to obtain the SPPS in (**c**). (**d**) Schematic showing the mechanism of the long-range FM interaction between two “A-vacancies”, which is mediated by the direct exchange interaction between the localized spins and the delocalized SPPS. (**e**) SPPS-mediated AFM interaction between two “vacancies” in opposite sublattices, which involves the superexchange interaction between the localized spins and the SPPS.

We next turn to the question of what mediates the long-range magnetic interaction in graphene. We temporarily ignore the spin degree of freedom and focus on the low-energy graphene states in the presence of a finite density of “A-vacancies”. Each “A-vacancy” induces a ‘zero-energy’ quasilocalized state, whose charge density is distributed only on the B sublattice sites^32^. The electron hopping between sites on the opposing sublattices of graphene then makes the “A-vacancy” state selectively hybridized with the $| {\rm{A}}\rangle$ sublattice state (Supplementary Fig. S1). As a result, the low-energy graphene state near the Fermi energy becomes “polarized” to the remaining $| {\rm{B}}\rangle$ pseudospin. We now consider the real spin polarization. Because their constituent orbitals are on the same sublattice, the direct exchange interaction between the spin-up “A-vacancy” state and the $| {\rm{B}}\rangle$ pseudospin induces parallel magnetization on the $| {\rm{B}}\rangle$ pseudospin. To demonstrate this effect, we considered two H_A_ impurities in a graphene supercell (Fig. 3a–c). For the FM state in Fig. 3a, the spin-up polarized $| {\rm{B}}\rangle$ (yellow) is induced as the spin-polarized pseudospin (SPPS), which fills the graphene (see Fig. 3c and legend for more details). The spin polarization of $| {\rm{B}}\rangle$ does not violate Lieb’s theorem^35^ because the local magnetic moments of the “A-vacancies” are reduced by the hybridization with $| {\rm{A}}\rangle$. We note that as a secondary effect, the spin-up $| {\rm{B}}\rangle$ induces the antiparallel magnetization (blue) on the opposite sublattice in Fig. 3a due to exchange polarization^18^. For the AFM state in Fig. 3b, however, the $| {\rm{B}}\rangle$ pseudospin is non-spin-polarized due to the spin up-down symmetry of the two “A-vacancies”, which results in spin polarization only around the impurities.

Figure 3d illustrates the mechanism of the SPPS-mediated FM interaction between the “vacancies” in the same sublattices. Here, we assume many “A-vacancies”, although only two of them are shown in the schematic. For the local magnetic moments $\{{m}_{i}^{A}\}$, $\sum _{i}{m}_{i}^{A} > 0$ was assumed; thus, the $| {\rm{B}}\rangle$ pseudospin is spin-up polarized. Regarding individual localized spins, each spin *m*^A^ interacts with the spin-up $| {\rm{B}}\rangle$ through the direct exchange interaction, which lowers the system’s energy when *m*^A^ takes the same spin direction as that of the SPPS (i.e., *m*^A^ = 1), with an energy gain proportional to ${J}_{LR}^{FM}\frac{{\sum }_{i}{m}_{i}^{A}}{|{\rm{\Lambda }}|}$. Therefore, the SPPS effectively mediates the pairwise FM interaction with the coupling strength of $\frac{{J}_{LR}^{FM}}{|{\rm{\Lambda }}|}$.

We now consider the mixture of H_A_ and H_B_ in graphene. The spin ground state has $\sum _{i}{m}_{i}^{A}=| {H}_{A}|$ and $\sum _{i}{m}_{i}^{B}=-\,| {H}_{B}|$ at zero temperature^18^, where |H_A_| and |H_B_| are the number of H adatoms at the graphene’s A and B sublattices. Assuming an antiparallel net magnetization on opposite sublattices at finite temperatures, the low-energy graphene state for the occupied electrons is characterized by the spin-up $| {\rm{B}}\rangle$ and spin-down $| {\rm{A}}\rangle$ (Supplementary Fig. S2). The spin-flipped counterparts constitute the unoccupied pseudospin state above the Fermi energy (Fig. 3e). In addition to the FM interaction between a pair of “vacancies” in the same sublattices, a superexchange interaction exists between the occupied (or unoccupied) “vacancy” state and the unoccupied (or occupied) SPPS of the same spin. The electron hopping between opposite sublattices results in the sublattice-dependent hybridization between the localized spins and the SPPS with the effective coupling $\alpha =\frac{{t}^{\ast }}{\sqrt{|{\rm{\Lambda }}|}}$, where t^*^ = 3.3 eV in the DFT calculations (Supplementary Fig. S1). The superexchange interaction is a second-order interaction proportional to 𝛼^2^ and thus scales with $\frac{1}{|{\rm{\Lambda }}|}$. For $\sum _{i}{m}_{i}^{A} > 0$ and $\sum _{i}{m}_{i}^{B} < 0$, the SPPS-mediated interaction energetically favors *m*^B^ = −1 and *m*^A^ = 1, hence effectively leading to the AFM interaction of $\frac{{J}_{LR}^{AFM}}{|{\rm{\Lambda }}|}$ for a pair of “vacancies” in opposing sublattices.

### Mean-field ferromagnetism in graphene

By combining the two types of SPPS-mediated interactions, we obtain the interaction energy for a given spin state $\{{m}_{i}^{A},\,{m}_{j}^{B}\}$,

3 $${E}_{LR}=-\,\frac{{J}_{LR}^{FM}}{2|{\rm{\Lambda }}|}\{\sum _{i,\,j}{m}_{i}^{A}{m}_{j}^{A}+\sum _{i,\,j}{m}_{i}^{B}{m}_{j}^{B}\}+\frac{{J}_{LR}^{AFM}}{|{\rm{\Lambda }}|}\sum _{i,\,j}{m}_{i}^{A}{m}_{j}^{B}.$$

**Figure 4:**
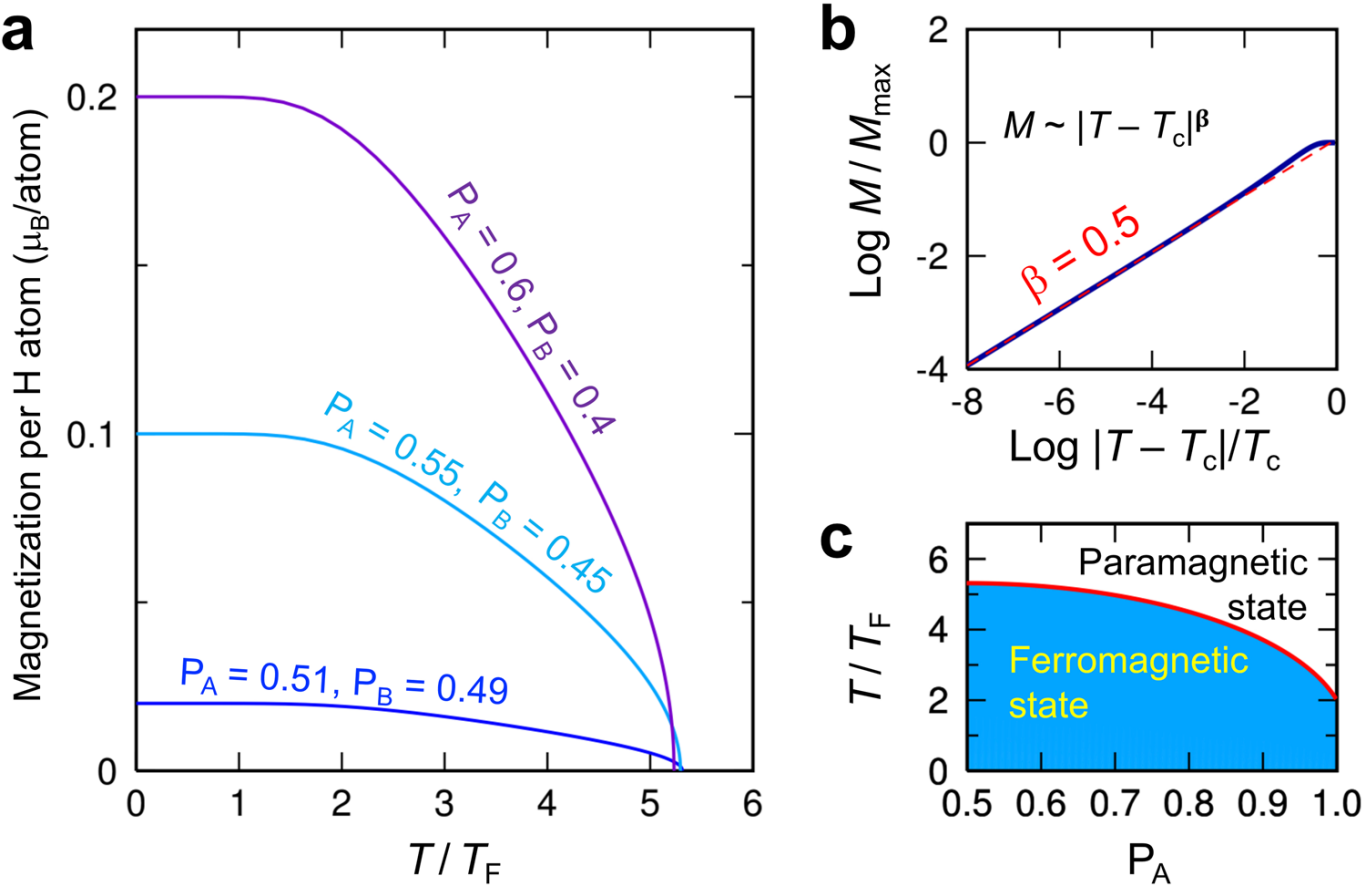
Mean-field ferromagnetism in graphene. (**a**) The simulated magnetization per H adatom as a function of the reduced temperature T/T_F_, where ${T}_{F}=\frac{{n}_{H}{J}_{LR}^{FM}}{4{k}_{B}}$. Different probabilities, P_A_ and P_B_, of having H_A_ and H_B_ on the graphene layer were considered. (**b**) Temperature dependence of the magnetization near the Curie temperature T_c_, indicating that the critical exponent β is 0.5. (**c**) The P_A_–T phase diagram of the hydrogenated graphene.

Our DFT calculations show that the AFM interaction is stronger than the FM interaction, with a ratio of $\frac{{J}_{LR}^{AFM}}{{J}_{LR}^{FM}}$ = 4.3 (see Methods for details). The long-range nature of the SPPS-mediated interactions allows us to use a mean-field approximation with ${E}_{LR}^{MFA}$ = $-(\tfrac{{J}_{LR}^{FM}|{H}_{A}|}{2|{\rm{\Lambda }}|}\langle {m}^{A}\rangle -\tfrac{{J}_{LR}^{AFM}|{H}_{B}|}{2|{\rm{\Lambda }}|}\langle {m}^{B}\rangle )\sum _{i}{m}_{i}^{A}$ − $(\tfrac{{J}_{LR}^{FM}|{H}_{B}|}{2|{\rm{\Lambda }}|}\langle {m}^{B}\rangle -\tfrac{{J}_{LR}^{AFM}|{H}_{A}|}{2|{\rm{\Lambda }}|}\langle {m}^{A}\rangle )\sum _{i}{m}_{i}^{B}$, in which the ensemble-averaged spins, 〈*m*^A^(T)〉 and 〈*m*^B^(T)〉, at temperature T are calculated self-consistently (Methods). We define two characteristic temperatures, ${T}_{F}=\frac{{n}_{H}{J}_{LR}^{FM}}{4{k}_{B}}$ and ${T}_{AF}=\frac{{n}_{H}{J}_{LR}^{AFM}}{4{k}_{B}}$, where k_B_ is the Boltzmann constant, and n_H_ is the atom percentage of H adatoms. Figure 4a shows the magnetization as a function of the reduced temperature T/T_F_ for the different probabilities, P_A_ and P_B_, of having H_A_ and H_B_ on the graphene layer. The magnetization per H adatom is $m=\frac{|{H}_{A}|-|{H}_{B}|}{|{H}_{A}|+|{H}_{B}|}={P}_{A}-{P}_{B}$ at T = 0, which is consistent with Lieb’s theorem^35^. A slight imbalance with P_A_ = 0.51 and P_B_ = 0.49, for example, induces *m* = 0.02 μ_B_/atom, which corresponds to the magnetization per weight of 0.1 Am^2^/kg at n_H_ = 1 at. % (Supplementary Fig. S3). The preferential H adsorption for one sublattice might be possible using a suitable substrate for graphene such as hexagonal boron nitride^36^ or exploiting the effect of the stacking order of multilayer graphene^18^. Near the Curie temperature T_c_, the magnetization M ~ (T_c_ – T)^β^ has the critical exponent β = 0.5, regardless of P_A_ and P_B_ (Fig. 4b), as expected from mean-field theory^37^.

Unlike the magnetization in Fig. 4a, the Curie temperature weakly depends on P_A_ and P_B_, which enables the enhanced magnetization while maintaining the high T_c_. The T_c_ is ${T}_{c}={T}_{F}+\sqrt{{({P}_{A}-{P}_{B})}^{2}{T}_{F}^{2}+4{P}_{A}{P}_{B}{T}_{AF}^{2}}$ and has a maximum at P_A_ = P_B_ with ${{T}_{c}}^{max}={T}_{F}+{T}_{AF}$ = n_H_ × 34,000 K (Fig. 4c). To achieve room-temperature ferromagnetism (Supplementary Fig. S3), it is necessary to introduce a relatively high concentration (~1 at. %) of H adatoms, which are magnetically active and not quenched by forming H-adatom “dimers”^11,18,34^. Partial hydrogenation of graphene with high n_H_ is challenging because of the phase separation of hydrogenated graphene^28^. Therefore, controlled hydrogenation under non-equilibrium conditions is required to realize room-temperature FM graphene.

We note that the AFM interaction contributes to ${{T}_{c}}^{max}={T}_{F}+{T}_{AF}$ as much as the FM interaction. This may appear to contradict the notion that the AFM interaction typically reduces the T_c_; for carrier-mediated FM semiconductors (e.g., Mn-doped GaAs), the T_c_ is determined by competition between the FM and AFM interactions^38^, i.e., ${T}_{c}={T}_{F}-{T}_{AF}$. For hydrogenated graphene, however, the SPPS-mediated FM and AFM interactions do not compete (Eq. 3); the spin-up $| {\rm{B}}\rangle$ pseudospin in Fig. 3e, for example, stabilizes the spin-up polarized H_A_ through the direct exchange interaction, and the same SPPS simultaneously stabilizes the spin-down H_B_ through the superexchange interaction. The FM and AFM interactions thus “cooperate”, rather than compete, in hydrogenated graphene.

## Summary and Conclusions

In summary, we have shown that the SPPS-mediated, long-range magnetic interactions give rise to a robust magnetic order in hydrogenated graphene, which can be stable even at room temperature for the H concentration of n_H_ ~ 1 at. %. The “cooperativeness” of the FM and AFM interactions contributes to the high T_c_. The realization of the robust 2D ferromagnetism in such a magnetically and electrically dilute system is unusual; the 2D magnetism in hydrogenated graphene is described by mean-field theory owing to the intrinsic long-range nature of the SPPS-mediated interactions. Our new finding of the mechanism underlying graphene ferromagnetism should have enormous implications for understanding and atomic-scale control of graphene-based magnetism, which is an important step toward bringing the vision described in “*Painting magnetism on a canvas of graphene* (ref.^12^)” closer to reality.

## Methods

### Spin-polarized density-functional theory (DFT) calculations

We calculated total energies and spin densities of partially hydrogenated graphene using the generalized gradient approximation (GGA-PBE^39^) to DFT, as implemented in the Vienna ab-initio Simulation Package^40^. The DFT calculations employed the projector augmented wave method^41,42^ with an energy cutoff of 500 eV for the plane-wave part of the wave function. For the Brillouin zone integration, we used a *Γ*-centred grid containing enough *k* points, as dense as the 60 × 60 grid for the two-atom unitcell of graphene.

### Calculations of the SPPS-mediated magnetic interactions

We first performed spin-polarized DFT calculations for H adatoms on the same sublattice of graphene to distinguish the long-range “mediated” FM interactions from the direct exchange interactions. To this end, the H positions in a graphene supercell were chosen so that only the direct pair interactions for the H pairs of a single type are involved in Eq. 1. The H pairs were selected among the three types of pairs denoted by pair 1, 2, and 3 in Fig. 2a. Different H concentrations were considered by changing either the number of H adatoms on a graphene supercell (*N*_H_ = 2, 3 or 4) or the number of the C atoms in a supercell with |*Λ*| = 96, 150, 216, 300, 384, 432, and 864 (see Supplementary Table S1 and legend for more details). Each data point in Fig. 2b was obtained from the energy difference for two different spin configurations that are listed in Supplementary Table S1. For the cases of *N*_flip_ = 0, we arbitrarily selected the “*N*_flip_ values” in ${\rm{\Delta }}{E}^{\ast }=\frac{{E}^{(1)}-{E}^{(2)}}{2{N}_{{\rm{flip}}}}$ and ${\rm{\Delta }}{M}^{\ast }=\frac{{({\sum }_{i}{m}_{i}^{A(1)})}^{2}-{({\sum }_{i}{m}_{i}^{A(2)})}^{2}}{4{N}_{{\rm{flip}}}|{\rm{\Lambda }}|}$ to plot the data in the same figure; note that these data also lie on a line with the y-intercept of zero as expected.

To extract the interaction strength of the long-range AFM interaction ${J}_{LR}^{{\rm{AFM}}}$, we considered two H adatoms on the opposing sublattices of the 864-atom graphene supercell. The two H adatoms in the supercell are well separated to ensure that the direct exchange interaction is negligible. Two spin configurations, {*m*^(1)^} = (up, down) and {*m*^(2)^} = (up, up), were considered. Then, from Eq. 3, the energy difference is ${E}_{LR}^{(1)}-{E}_{LR}^{(2)}=-\,\frac{2{J}_{LR}^{AFM}}{|{\rm{\Lambda }}|}$, which was calculated from the spin-polarized DFT calculations to determine ${J}_{LR}^{{\rm{AFM}}}$.

### Finite-temperature magnetism of hydrogenated grapheme

We used a mean-field approximation with ${E}_{LR}^{MFA}$ = $-(\tfrac{{J}_{LR}^{FM}|{H}_{A}|}{2|{\rm{\Lambda }}|}\langle {m}^{A}\rangle -\tfrac{{J}_{LR}^{AFM}|{H}_{B}|}{2|{\rm{\Lambda }}|}\langle {m}^{B}\rangle )\sum _{i}{m}_{i}^{A}$ − $(\tfrac{{J}_{LR}^{FM}|{H}_{B}|}{2|{\rm{\Lambda }}|}\langle {m}^{B}\rangle -\tfrac{{J}_{LR}^{AFM}|{H}_{A}|}{2|{\rm{\Lambda }}|}\langle {m}^{A}\rangle )\sum _{i}{m}_{i}^{B}$ to obtain the ensemble averaged spins at temperature T. We introduced two characteristic temperatures, ${T}_{F}=\frac{{n}_{H}{J}_{LR}^{FM}}{4{k}_{B}}$ and ${T}_{AF}=\frac{{n}_{H}{J}_{LR}^{AFM}}{4{k}_{B}}$, where k_B_ is the Boltzmann constant, and n_H_ is the H concentration, ${n}_{H}=\frac{|{H}_{A}|+|{H}_{B}|}{|\Lambda |}$. For ${P}_{A}=\frac{|{H}_{A}|}{|{H}_{A}|+|{H}_{B}|}$ and ${P}_{B}=\frac{|{H}_{B}|}{|{H}_{A}|+|{H}_{B}|}$, the coupled equations for $\langle {m}^{{\rm{A}}}\rangle$ and $\langle {m}^{{\rm{B}}}\rangle$ are given by $\langle {m}^{A}\rangle =\,\tanh [\frac{2}{T}({T}_{F}{P}_{A}-{T}_{AF}{P}_{B}\eta (T)){\langle m\rangle }^{A}]$ and $\langle {m}^{B}\rangle =\,\tanh [\frac{2}{T}({T}_{F}{P}_{B}-{T}_{AF}{P}_{A}\frac{1}{\eta (T)})\langle {m}^{B}\rangle ]$, where $\eta (T)$ is the ratio of the magnetization on the opposing sublattices. $\eta (T)$ was determined numerically as a function of temperatures. We found that $\eta$ is always negative; for P_A_ = P_B_, $\eta$ is constant with $\eta =-\,1$, while for the case of P_A_ ≠ P_B_, it gradually increases in magnitude with increasing T. From the condition of no net magnetization on each sublattice at T_c_, we obtained $\eta ({T}_{c})=-\frac{1}{2}\frac{{T}_{F}}{{T}_{AF}}(1-\frac{{P}_{A}}{{P}_{B}})-$ $\sqrt{\frac{{P}_{A}}{{P}_{B}}+\frac{1}{4}{(\frac{{T}_{F}}{{T}_{AF}})}^{2}{(1-\frac{{P}_{A}}{{P}_{B}})}^{2}}$ and ${T}_{c}={T}_{F}+\sqrt{{({P}_{A}-{P}_{B})}^{2}{T}_{F}^{2}+4{P}_{A}{P}_{B}{T}_{AF}^{2}}$.

## Electronic supplementary material

Supplementary Information
