# Supplementary material for: Robust ferromagnetism in hydrogenated graphene mediated by spin-polarized pseudospin
Source: Sci Rep. 2018 Sep 17;8:13940. doi: 10.1038/s41598-018-31934-0 (PMC6141587; doi:10.1038/s41598-018-31934-0)
Supplement: Supplementary file 1 — Supplementary Information [file 41598_2018_31934_MOESM1_ESM.pdf]

Supplementary Information for

Robust ferromagnetism in hydrogenated graphene mediated by spin-polarized pseudospin

Hyunyoung Kim[1], Junhyeok Bang[2] & Joongoo Kang[1,3*]

[1]Department of Emerging Materials Science, DGIST, Daegu 42988, Korea

[2]Spin Engineering Physics Team, Korea Basic Science Institute (KBSI), Daejeon 305-806, Korea

[3]Center for Bio-Convergence Spin System, DGIST, Daegu 42988, Korea

*Corresponding author: joongoo.kang@dgist.ac.kr

This PDF file includes:

- Table S1
- Figures S1-S3

Table S1. Selected hydrogenated graphene systems for the DFT calculations in Fig. 2. $|\Lambda|$ denotes the number of C atoms in a graphene supercell and N_{H} is the number of H adatoms in a supercell. The H adatoms in each graphene supercell exist either as an isolated H or as an H pair of a single type, which was chosen among the three types of pairs denoted by pair 1, 2, and 3 in the inset of Fig. 2a. For N_{H} = 2, the H pair of a given type involves the two localized spins at the adsorption sites, each having a spin direction denoted by "u" for spin up or "d" for spin down. For N_{H} = 3, one H adatom exists as an isolated adatom, while the other two H adatoms exist as a pair. For example, the spin configuration (u, ud) represents a single H adatom with the spin-up magnetic moment and an AFM-coupled pair. For N_{H} = 4, two H pairs of the same type were considered for different spin configurations. N_{flip} is the number of the FM-to-AFM spin flips involved in the change from $\{m_i^{(1)}\}$ to $\{m_i^{(2)}\}$. The energy differences of the two spin configurations are listed. For each data point in Fig. 2b, the corresponding symbol is listed in the last column of the table.

H pair	$\|\Lambda\|$	N_{H}	$\{m_i^{(1)}\}$	$\{m_i^{(2)}\}$	$\sum m_i^{(1)}$	$\sum m_i^{(2)}$	N_{flip}	$E^{(1)} - E^{(2)}$ (eV)	
**Pair 1**	96	2	(uu)	(ud)	2	0	1	-0.126	○
	150		(uu)	(ud)	2	0	1	-0.110	○
	216		(uu)	(ud)	2	0	1	-0.101	○
	384		(uu)	(ud)	2	0	1	-0.090	○
	864		(uu)	(ud)	2	0	1	-0.079	○
	300	3	(u, uu)	(u, ud)	3	1	1	-0.106	○
	432		(d, uu)	(d, ud)	1	-1	1	-0.081	○
	300	4	(uu, uu)	(ud, uu)	4	2	1	-0.124	○
	300		(uu, ud)	(ud, ud)	2	0	1	-0.095	○
	300		(uu, uu)	(ud, ud)	4	0	2	-0.219	○
	300		(uu, dd)	(ud, dd)	0	-2	1	-0.062	○
	300		(uu, dd)	(ud, ud)	0	0	2	-0.157	○
	300		(uu, uu)	(uu, dd)	4	0	0	-0.062	●
	432		(uu, uu)	(ud, uu)	4	2	1	-0.110	○
	432		(uu, ud)	(ud, ud)	2	0	1	-0.089	○
	432		(uu, uu)	(ud, ud)	4	0	2	-0.199	○

	432		(uu, dd)	(ud, dd)	0	-2	1	-0.071	○
	432		(uu, dd)	(ud, ud)	0	0	2	-0.160	○
	432		(uu, uu)	(uu, dd)	4	0	0	-0.040	●
**Pair 2**	96	2	(uu)	(ud)	2	0	1	-0.086	□
	150		(uu)	(ud)	2	0	1	-0.068	□
	216		(uu)	(ud)	2	0	1	-0.060	□
	384		(uu)	(ud)	2	0	1	-0.051	□
	432		(uu)	(ud)	2	0	1	-0.051	□
	864		(uu)	(ud)	2	0	1	-0.043	□
	300	3	(u, uu)	(u, ud)	3	1	1	-0.068	□
	300		(d, uu)	(d, ud)	1	-1	1	-0.037	□
	432		(u, uu)	(u, ud)	3	1	1	-0.059	□
	432		(d, uu)	(d, ud)	1	-1	1	-0.038	□
	300	4	(uu, uu)	(ud, uu)	4	2	1	-0.080	□
	300		(uu, ud)	(ud, ud)	2	0	1	-0.054	□
	300		(uu, uu)	(ud, ud)	4	0	2	-0.135	□
	300		(uu, dd)	(ud, dd)	0	-2	1	-0.026	□
	300		(uu, dd)	(ud, ud)	0	0	2	-0.081	□
	300		(uu, uu)	(uu, dd)	4	0	0	-0.054	■
	432		(uu, uu)	(ud, uu)	4	2	1	-0.068	□
	432		(uu, ud)	(ud, ud)	2	0	1	-0.050	□
	432		(uu, uu)	(ud, ud)	4	0	2	-0.118	□
	432		(uu, dd)	(ud, dd)	0	-2	1	-0.029	□
	432		(uu, dd)	(ud, ud)	0	0	2	-0.079	□
	432		(uu, uu)	(uu, dd)	4	0	0	-0.039	■
**Pair 3**	150	2	(uu)	(ud)	2	0	1	-0.048	△
	216		(uu)	(ud)	2	0	1	-0.037	△
	384		(uu)	(ud)	2	0	1	-0.028	△
	432		(uu)	(ud)	2	0	1	-0.029	△
	864		(uu)	(ud)	2	0	1	-0.022	△
	300	3	(u, uu)	(u, ud)	3	1	1	-0.048	△
	300		(d, uu)	(d, ud)	1	-1	1	-0.018	△
	432		(u, uu)	(u, ud)	3	1	1	-0.039	△
	432		(d, uu)	(d, ud)	1	-1	1	-0.017	△
	300	4	(uu, uu)	(ud, uu)	4	2	1	-0.061	△
	300		(uu, ud)	(ud, ud)	2	0	1	-0.033	△
	300		(uu, uu)	(ud, ud)	4	0	2	-0.094	△

	300		(uu, dd)	(ud, dd)	0	-2	1	-0.005	△
	300		(uu, dd)	(ud, ud)	0	0	2	-0.038	△
	300		(uu, uu)	(uu, dd)	4	0	0	-0.056	▲
	432		(uu, uu)	(ud, uu)	4	2	1	-0.047	△
	432		(uu, ud)	(ud, ud)	2	0	1	-0.027	△
	432		(uu, uu)	(ud, ud)	4	0	2	-0.074	△
	432		(uu, dd)	(ud, dd)	0	-2	1	-0.008	△
	432		(uu, dd)	(ud, ud)	0	0	2	-0.035	△
	432		(uu, uu)	(uu, dd)	4	0	0	-0.039	▲

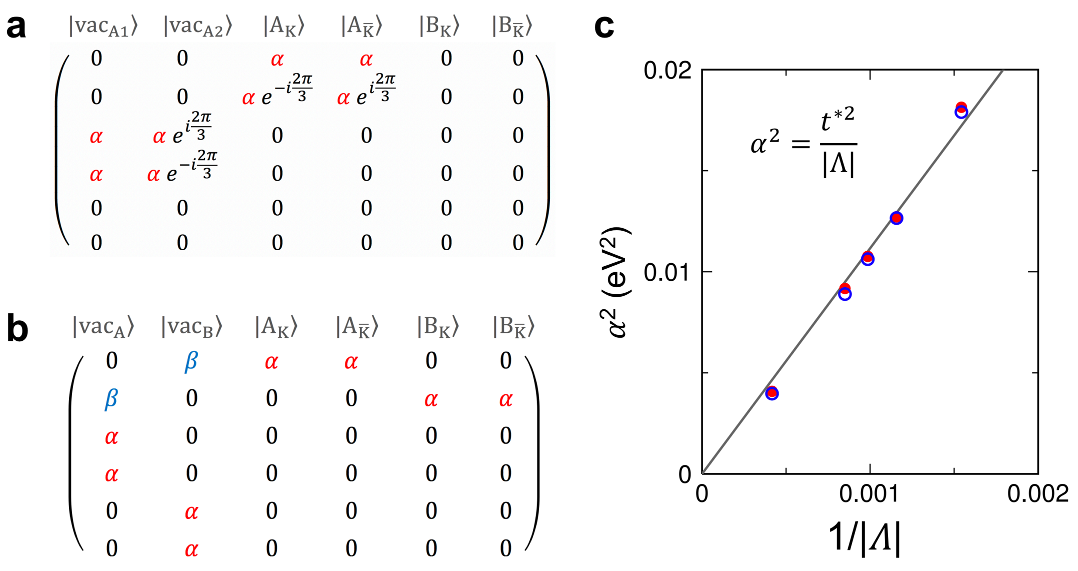

Figure S1. Low-energy effective Hamiltonians. (**a**) Effective Hamiltonian for the two H adatoms (or "vacancies") on the same sublattice A of a graphene supercell. The atomic concentration of H adatoms is thus $n_H = \frac{2}{|\Lambda|}$. We first ignore the spin degree of freedom and focus on the effect of "vacancies" on the low-energy graphene states. To allow intervalley scattering in the calculations, a supercell is constructed so that the two valleys at K and –K are folded to the Γ point of the Brillouin zone of a supercell. The electron hopping between sites on the opposing sublattices of graphene makes the "A-vacancy" state selectively hybridized with the $|A\rangle$ sublattice state at each valley. The size of the coupling strength is $|\alpha|$, and the phase factor of $e^{i\frac{2\pi}{3}}$ (or $e^{-i\frac{2\pi}{3}}$) originates from the Bloch phase difference of $|A_K\rangle$ (or $|A_{\bar{K}}\rangle$) at the selected H-adatom positions. (**b**) Same as (**a**) but for the two H adatoms on opposing sublattices of a graphene supercell. The sublattice state $|A\rangle$ (or $|B\rangle$) at each valley is hybridized with the "A-vacancy" (or "B-vacancy") state in the supercell, with the corresponding matrix element α. In addition, the hopping matrix element β between the "A-vacancy" and "B-vacancy" states exist, and it decreases as the separation between the H adatoms increases. (**c**) Using non-spin-polarized DFT calculations, the coupling strength α in the Hamiltonian in (**a**) was determined from the non-zero eigenvalues of $\pm\alpha$ and $\pm\sqrt{3}\alpha$, as

a function of $1/|\Lambda|$ (red filled circles). Likewise, the coupling strength α in (**b**), as well as β, was calculated from the non-zero eigenvalues of $\frac{\beta}{2} \pm \sqrt{2\alpha^2 + \frac{\beta^2}{4}}$ and $-\frac{\beta}{2} \pm \sqrt{2\alpha^2 + \frac{\beta^2}{4}}$ (blue open circles). In both cases, we obtained almost the same α for a given supercell size. The linear dependence of α^2 on $\frac{1}{|\Lambda|}$ indicates that α is given by $\alpha = \frac{t^*}{\sqrt{|\Lambda|}}$. From the slope in **c**, the effective electron hopping t^* was calculated to be $t^* = 3.3$ eV.

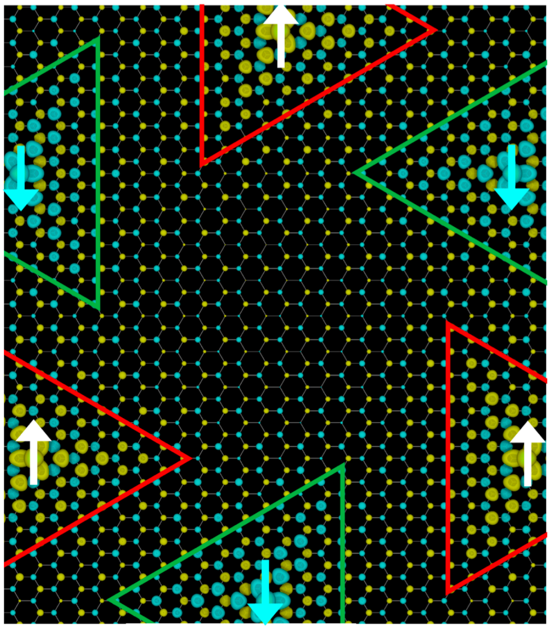

Figure S2. Spin-polarized pseudospin (SPPS) of hydrogenated graphene. Spin density of the 864-atom graphene supercell containing two H adatoms on the opposing sublattices. The spin density of the expanded supercell is shown for the spin-up (yellow) and spin-down (blue) electrons at 0.0016 $|e|/Å^3$. H_A is spin-up polarized, while H_B is spin-down polarized. The SPPS is then characterized by the spin-up $|B\rangle$ and spin-down $|A\rangle$.

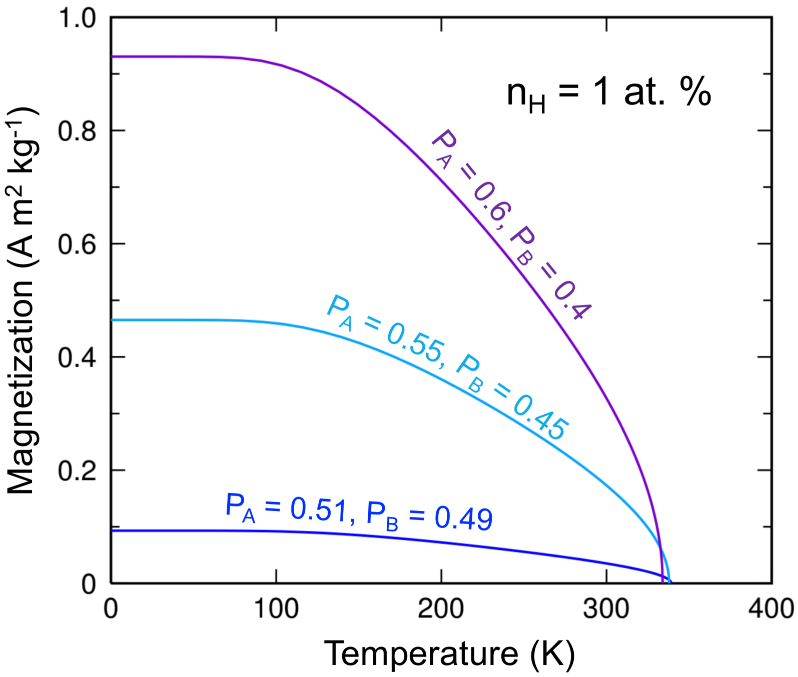

Figure S3. Room-temperature ferromagnetic graphene. The simulated magnetization per weight as a function of temperatures for the H concentration of n_H = 1 at. %. Different probabilities, P_A and P_B, of having H_A and H_B on the graphene layer were considered.
